# E2F1 facilitates DNA break repair by localizing to break sites and enhancing the expression of homologous recombination factors

**DOI:** 10.1038/s12276-019-0307-2

**Published:** 2019-09-18

**Authors:** Eui-Hwan Choi, Keun Pil Kim

**Affiliations:** Department of Life Sciences, Chung-Ang University, Seoul, 06974 South Korea

**Keywords:** Cancer genetics, Cell death, Genomic instability

## Abstract

The human genome is constantly exposed to both endogenous and exogenous stresses, which can lead to errors in DNA replication and the accumulation of DNA mutations, thereby increasing the risk of cancer development. The transcription factor E2F1 is a key regulator of DNA repair. E2F1 also has defined roles in the replication of many cell cycle-related genes and is highly expressed in cancer cells, and its abundance is strongly associated with poor prognosis in cancers. Studies on colon cancer have demonstrated that the depletion of E2F1 leads to reduced levels of homologous recombination (HR), resulting in interrupted DNA replication and the subsequent accumulation of DNA lesions. Our results demonstrate that the depletion of E2F1 also causes reduced RAD51-mediated DNA repair and diminished cell viability resulting from DNA damage. Furthermore, the extent of RAD51 and RPA colocalization is reduced in response to DNA damage; however, RPA single-stranded DNA (ssDNA) nucleofilament formation is not affected following the depletion of E2F1, implying that ssDNA gaps accumulate when RAD51-mediated DNA gap filling or repair is diminished. Surprisingly, we also demonstrate that E2F1 forms foci with RAD51 or RPA at DNA break sites on damaged DNA. These findings provide evidence of a molecular mechanism underlying the E2F1-mediated regulation of HR activity and predict a fundamental shift in the function of E2F1 from regulating cell division to accelerating tumor development.

## Cancer: Covering up DNA damage

A protein that helps repair damaged DNA, E2F1, paradoxically also promotes tumor growth. Before cells can divide, their DNA must be copied. DNA damage, as occurs in cancer cells, often interrupts DNA copying, like a missing tooth in a zipper; such cells then self-destruct. Puzzlingly, most cancer cells show high levels of E2F1, which is supposed to repair damaged DNA. Keun Pil Kim and Eui-Hwan Choi at Chung-Ang University in Seoul, South Korea investigated how this molecule regulates DNA repairs in cancer cells. They found that cancer cells without E2F1 could not copy their damaged DNA, and self-destructed. Further study showed that E2F1 responded to sites of DNA damage, recruiting other DNA repair factors, permitting the cells to replicate. These results suggest new targets in the fight against cancer.

## Introduction

The proliferation of tumor cells is specifically associated with genomic instability, the subsequent increased mutation rate and the acceleration of tumorigenesis^1^. Deregulation of DNA replication, which is generally described as replication stress, accounts for a portion of this cellular disorder. In addition, responses to DNA damage, which are often regulated by robust cellular mechanisms, are capable of both inducing checkpoint arrest and stimulating DNA repair, thus maintaining general genomic integrity. In cancer cells, however, owing to aberrant DNA repair responses and constitutive growth signaling, “replication stress”, a unique phenomenon in cancer cells that prevents error-free DNA replication and delays DNA synthesis, may occur^1–6^. Furthermore, oncogenes induced by inappropriate cellular replication drive genomic instability and contribute to the discontinuous process of the replication fork, replication-transcription discord, and defects in nucleotide metabolism^2,3^. Incomplete DNA replication can also lead to the accumulation of gene mutations or DNA gaps, which can cause the breakage, missegregation, and rearrangement of chromosomes^7–11^. Thus, multiple conditions, including those resulting in DNA damage, may act to inhibit DNA replication and interrupt cellular progression, thereby causing genome instability^2,12,13^.

The E2F family of proteins is a group of transcription factors that regulate the activation of gene expression significant for DNA replication, DNA repair, and the control of cell cycling^14–18^. E2F1 has been described as responsive to specific cell cycle phases and DNA damage^18–21^. Furthermore, E2F1 is reported to be overexpressed in specific cancer cells compared with its expression in normal cells, and the upregulation of E2F1 has been associated with poor prognosis^16,22–24^. Recently, several studies have provided an evidence suggesting a role for the E2F proteins as biomarkers in cancers^24,25^. In addition, in a study that included 165 lymph node-negative breast carcinomas, patients with E2F1-positive tumors demonstrated reduced overall survival as well as reduced disease-free survival rates compared with those in patients with E2F1-negative tumors^26^. Although these results suggest that E2F1 is a dependable marker for multiple cancer types, its expression level within these cancers, specific biological function and molecular mechanism of action remain mostly unknown.

Another group of proteins, the Rad proteins, are also involved in DNA repair. Specifically, RAD51, RAD52, and RAD54 have been designated as the key proteins in homologous recombination (HR). These proteins play a central role in strand exchange between an undamaged homolog and a double-strand break (DSB) end, resulting in repair of the damaged regions. Furthermore, Rad protein levels are significantly elevated (by ~2–7-fold) in primary tumors and various cancer cell lines^27,28^. The advantage of the elevated expression of RAD51 for tumor cells has yet to be sufficiently explained; however, it has been suggested that overexpression of RAD51 is related to tumor progression caused by genomic disruption^27,29^. An alternate explanation is that elevated levels of RAD51 may provide cells with an advantage during DNA replication within rapid cell division^30,31^.

In this study, we show that E2F1 regulates gene expression for HR factors and DNA replication. We also demonstrate an essential role for the HR pathway in colon cancer progression. This pathway plays a role in the maintenance of genomic integrity and overcoming replication stress and thus acts to maintain genomic integrity during DNA replication and cell proliferation within colon cancer cells.

We first described the expression patterns of genes involved in DNA synapsis and synthesis through qPCR analysis. The expression of genes involved in the search for DNA homologs, DNA strand exchange, DNA replication and chromatin remodeling were altered following the depletion of E2F1. Second, we examined whether E2F1-mediated HR activity is essential to maintain the efficacy and fidelity of cellular progression. We found that HR factors were constitutively expressed in colon cancer cells. Therefore, these factors may enable the more rapid progression of replication forks and prevent accumulation of DNA breaks, including single-strand DNA (ssDNA) gaps in S-phase, by postreplication repair (PRR). In addition, cancer cells may utilize the HR pathway to maintain cellular proliferation and genomic integrity. Third, we demonstrated that the depletion of E2F1 leads to the reduced expression of HR factors, causing cell cycle arrest and cell death. Thus, the loss of these cellular processes due to functional defects in HR factors is an important event leading to genomic instability. Finally, we investigated the intracellular localization of E2F1, RAD51, and replication protein A (RPA) in colon cancer cells exposed to methyl methanesulfonate (MMS), which induces DNA double-strand breaks (DSBs). In cells experiencing DNA damage, the number of RAD51 and RPA foci increased, suggesting that 3′-single-stranded DNAs were exposed. Surprisingly, E2F1 proteins were localized at sites of DNA breakage along with γH2AX, RAD51, and RPA. These findings suggest that E2F1 is involved in a DNA repair mechanism with other DNA repair proteins and that these proteins form foci at broken DNA or ssDNA gaps. Furthermore, E2F1 depletion reduced the formation of RAD51 foci, but not the formation of RPA and γH2AX foci, and the accumulation of ssDNA gaps, indicating that colon cancer cells are able to undergo typical replication processes in the presence of unrepaired ssDNA gaps. This finding supports the possibility that unrepaired ssDNA gaps induce cell cycle arrest at the S/G2 phase. Based on these findings, we suggest a novel mechanism for the involvement of genomic instability in tumorigenesis that is regulated by E2F1-induced activation of the HR pathway and potential novel target genes for the inhibition of tumorigenesis.

## Materials and methods

### Cell culture

The HCT116 and HT29 human colon cancer cell lines, which were a kind gift from Hwang D.S., Ph.D., were cultured in Dulbecco’s modified Eagle medium (DMEM; Cat. No. 11995-073; Gibco) with heat-inactivated 10% fetal bovine serum (FBS; Cat. No. 16000-044; Gibco) and 100 U/ml penicillin/streptomycin (Cat. No. 15140-122; Gibco). The cells were maintained in an incubator at 37 °C and 5% CO_2_.

### Immunoprecipitation and immunoblot analysis

Cell lysates from HCT116 and HT29 human cell lines were prepared as described previously^32^. Whole-cell lysates (800 μg) were prepared for immunoprecipitation with protein A/G-agarose beads (Cat. No. P9203; GenDEPOT) according to the manufacturer’s instructions. Samples were incubated with primary antibodies at 4 °C. Following incubation, the samples were incubated with protein A/G-agarose beads for 3 h at 4 °C. The samples were then boiled and subjected to polyacrylamide gel electrophoresis for immunoblotting. The protein levels on the PVDF membrane were observed using a ChemiDoc^TM^ MP imaging system (Bio-Rad, Hercules, CA, USA). Antibodies specific for RAD54 (Cat. No. sc-374598, 1:1000), RAD51 (Cat. No. sc-8349, 1:3000), E2F1 (Cat. No. sc-251, 1:2000) and PCNA (Cat. No. sc-56, 1:2000) were purchased from Santa Cruz Biotechnology (USA). Antibody specific for Flag (Cat. No. F3165, 1:2000) was purchased from Sigma-Aldrich (USA), antibody specific for RPA2 (Cat. No. #2208, 1:3000) was purchased from Cell Signaling Technology (USA) and antibody specific for GAPDH (Cat. No. ab8245, 1:10,000), which was used as a housekeeping gene, was purchased from Abcam (UK).

### Quantitative PCR

Total RNA was isolated using the RNeasy Mini Kit (Cat. No. 74104; Qiagen), according to the manufacturer’s instructions. One microgram of total RNA from HCT116 and HT29 cells was reverse transcribed using a MicroRNA Reverse Transcription Kit (Cat. No. 4366596; Applied Biosystems). Quantification of cDNA by quantitative (q)PCR was performed using SYBR Green (Cat. No. K-6251; Bioneer) and a Bio-Rad Real-Time (RT)-PCR system. Oligo information is listed in Supplementary Table S1.

### BrdU incorporation assay

To analyze DNA replication, bromodeoxyuridine (BrdU) (Cat. No. B5002; Sigma Corp.) was added to the cell growth medium at a final concentration of 10 μM for 15 min. BrdU-labeled cells were washed with PBS, harvested, and fixed with 70% cold ethanol for 1 h at 4 °C. The fixed cells were treated with buffer (0.5% Triton X-100 and 2 M HCl in PBS) for 25 min. Cells were then washed with PBS and treated with neutralization buffer (0.1 M Na_2_B_4_O_7_–H_10_O_2_, pH 8.5) for recovery followed by incubation with anti-BrdU antibody (Cat. No. ab6326; Abcam, UK) for 1 h at 25 °C. Cells were then stained with a FITC-conjugated secondary antibody and propidium iodide (PI). Finally, the cell cycle was analyzed using flow cytometry (BD FACSCalibur).

### Comet assay

Human colon cancer cells were harvested and suspended in 300 μl of 1% low-melting temperature agarose gel (42 °C) (Cat. No. 50101; TaKaRa) at a cell density of 2 × 10^4^ cells/ml. The agarose gel was pipetted onto glass slides. Slides were incubated in lysis buffer (0.5 M Na_2_-EDTA, 0.5 mg/ml proteinase K (pH 8.0), 2% sodium lauroyl sarcosinate) overnight at 37 °C. The slides were then washed in rinse buffer (90 mM Tris, 90 mM boric acid, 2 mM Na_2_EDTA (pH 8.5)) for 30 min. Electrophoresis was also conducted in rinse buffer for 20 min at 20 V/19 cm. The slides were immersed in rinse buffer for 10 min, stained with PI staining solution for 10 min and observed using fluorescence microscopy. Comets were quantified using CASP software (1.2.3beta2).

### Measurement of apoptosis

To analyze cell apoptosis, human colon cancer cells were harvested and incubated in Annexin V-FITC binding buffer (10 mM HEPES (pH 7.4), 150 mM NaCl, 2.5 mM CaCl_2_). Annexin V-FITC (Cat. No. LS-02-100; BioBud) was then added to the suspended cells at a final concentration of 1.5 μg/ml and incubated for 15 min. Labeled cells were washed with cold 1 × PBS to remove excess Annexin V. The cells were stained with PI at a final concentration of 20 μg/ml and analyzed using flow cytometry (BD FACSCalibur). Quantification of FACS data was performed using FlowJo software.

### Immunofluorescence staining

Cells were attached to coverslips coated with 1 × poly-L-lysine and fixed with cold 4% paraformaldehyde. Briefly, the cells were treated with Triton X-100 (0.2%) for 5 min and blocked with blocking buffer (0.02% Tween-20 and 1% BSA in PBS) for 30 min. The preparations were incubated for 1 h with the following primary antibodies: anti-RAD51, anti-RPA, and anti-γH2AX. Following three washes with PBS-T (0.02% Tween-20 in PBS), the preparations were incubated for 50 min with the following secondary fluorescent antibodies: anti-Alexa 488 and anti-Cy3. After three washes with PBS-T, the preparations were mounted with mounting solution containing 2 μg/ml 4′,6-diamidino-2-phenylindole (DAPI). Images were obtained using a Nikon Eclipse Ti-E fluorescence microscope with a ×100 lens (NA = 1.49) and ×100 oil objective (ND = 1.515; Cat. No. 107590; Nikon).

### RNA interference

Commercially available AccuTarget^TM^ small interfering RNA (siRNA) specific for the *E2F1* gene (si*E2F1*) was used to knockdown any endogenous E2F1 expression in human colon cancer cells. The siRNA pool included single targeting oligonucleotides with the following sequence: 5′-GCUAUGAGACCUCACUGAA(dTdT)-3′. The oligonucleotides were transfected using DharmaFECT (Cat. No. T-2001; Dharmacon) according to the manufacturer’s instructions. A nontargeting siRNA (siControl) was used as a negative control (Cat. No. D-001810-10-05; Dharmacon). The cells were treated with the siRNAs in DMEM medium for 48 h and harvested.

## Results

### Depletion of E2F1 suppresses the expression of genes involved in HR and DNA replication

**Fig. 1:**
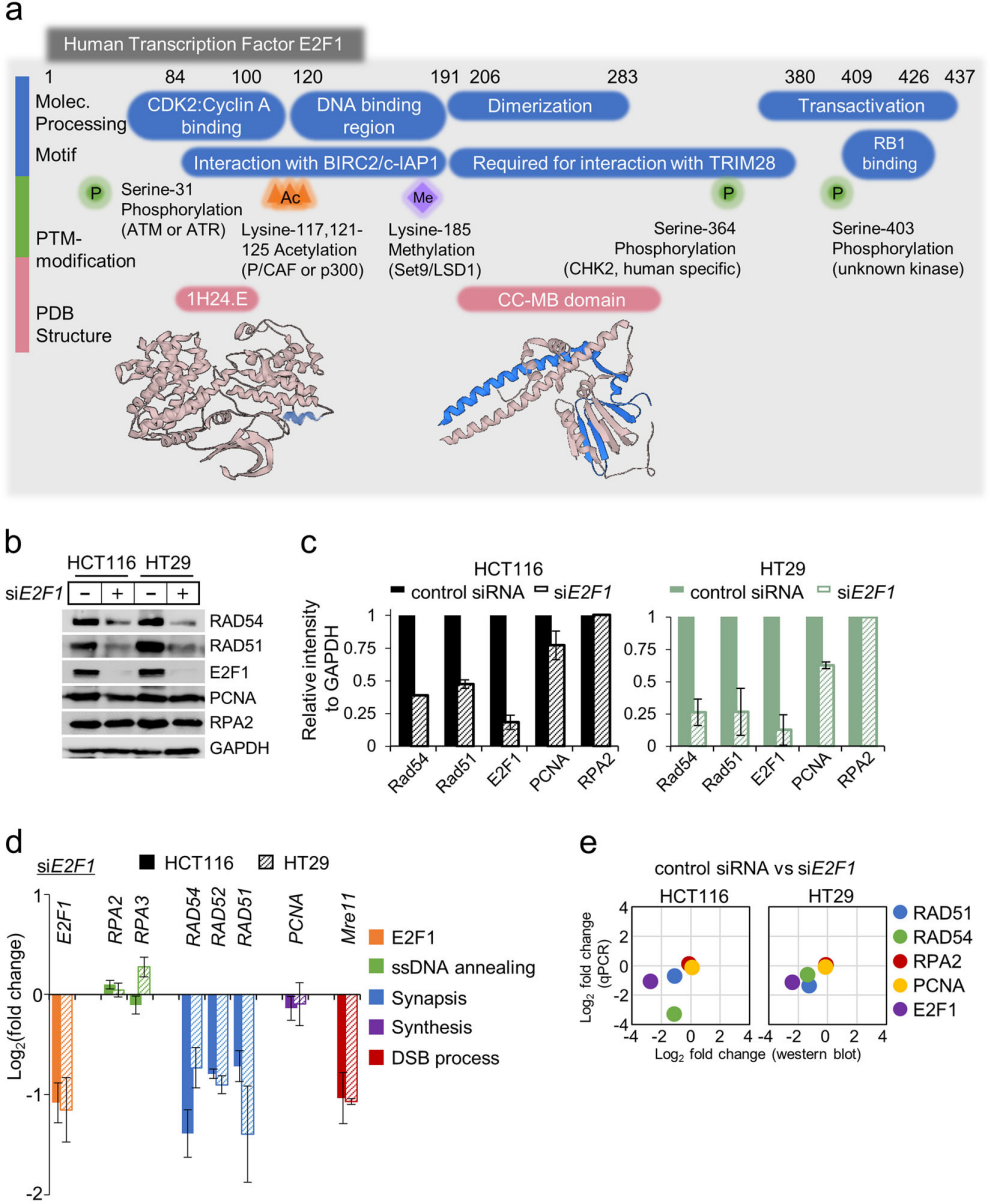
E2F1 regulates the expression of multiple factors involved in DNA repair, replication, and recombination. **a** Conserved domains of E2F1. E2F1 contains a cyclin A-binding domain including nuclear localization signals, a heptad repeat, marked box and a transactivation domain including pRB-binding regions^34^. The coordinates for the E2F1 protein structures described in this study have been deposited in the Protein Data Bank under ID codes 1H24E and 2AZE^33^. **b** Immunoblot analysis of whole-cell lysate extracts prepared from HCT116 and HT29 cells. The cells were transfected with siRNA against *E2F1* (si*E2F1*) or nontargeting siRNA (siControl) for 48 h. GAPDH was used as a housekeeping gene. **c** The expression levels of proteins in (**a**) were quantified, and the ratio relative to 18 s ribosomal RNA expression was determined for each condition. Each sample was normalized to the siControl condition. Three independent experiments were performed. Error bars denote the mean ± SD (*n* = 3). **d** Quantitative PCR analysis of changes in the expression of multiple genes in response to *E2F1* knockdown. The expression of synapsis- and synthesis-related genes, but not ssDNA annealing-related genes, was substantially reduced in cells transfected with si*E2F1*. Three independent qPCR experiments were performed. Error bars denote the mean ± SD (*n* = 3). The fold-change value for each sample was normalized to the siControl condition. **e** Comparison of changes in the expression of specific HR factors following *E2F1* knockdown, as assessed by immunoblot analysis and qPCR

E2F1 contains several conserved domains, including a cyclin A-binding domain, a heptad repeat, a DNA-binding domain and a transactivation domain with a retinoblastoma protein (pRB)-binding region^33,34^ (Fig. 1a). To identify the specific roles of E2F1 in the transcriptional regulation of human colon cancer cells, we examined the expression levels of HR factors following the knockdown of E2F1. In colon cancer cells, E2F1 and specific HR proteins had accumulated at higher levels (increased by 8.9/5.7-fold for E2F1, 8.7/7.6-fold for RAD51, 11.4/10.8-fold for RAD54, and 5.9/5.1-fold for RPA in HCT116/HT29 cells, respectively) than those in normal colon cells. These results support the possibility that colon cancers require abundant HR proteins to enhance HR-regulated DNA repair, which is essential for the maintenance of genomic integrity (Supplementary Fig. S1). To characterize the cellular dynamics of E2F1, we analyzed the expression level of multiple genes involved in DNA replication and the HR pathway in the presence or absence of E2F1. Interestingly, in cells treated with si*E2F1*, the expression of *RAD51* and *RAD54* was downregulated. However, the absence of E2F1 did not significantly impact the expression of RPA and PCNA compared with that in normal control cells (Fig. 1b). These results suggest that E2F1 activity is related to the regulation of HR gene expression in colon cancer cells and that low levels of E2F1 may thus lead to the suppression of the HR pathway (Fig. 1b, c). To investigate E2F1-mediated regulation of HR gene expression in colon cancers, we analyzed mRNA expression levels in colon cancer cells in the presence or absence of si*E2F1* via qPCR. Genes involved in the regulation of HR progression were classified into four groups: ssDNA annealing, synapsis, synthesis, and DSB processing genes (Fig. 1d). The levels of transcripts involved in the HR pathway were significantly decreased in cells following E2F1 knockdown compared with their expression in normal control cells; however, the expression of ssDNA annealing genes was not impacted by E2F1 knockdown (Fig. 1d, e).

### Depletion of E2F1 induces cell death in colon cancer cells

**Fig. 2:**
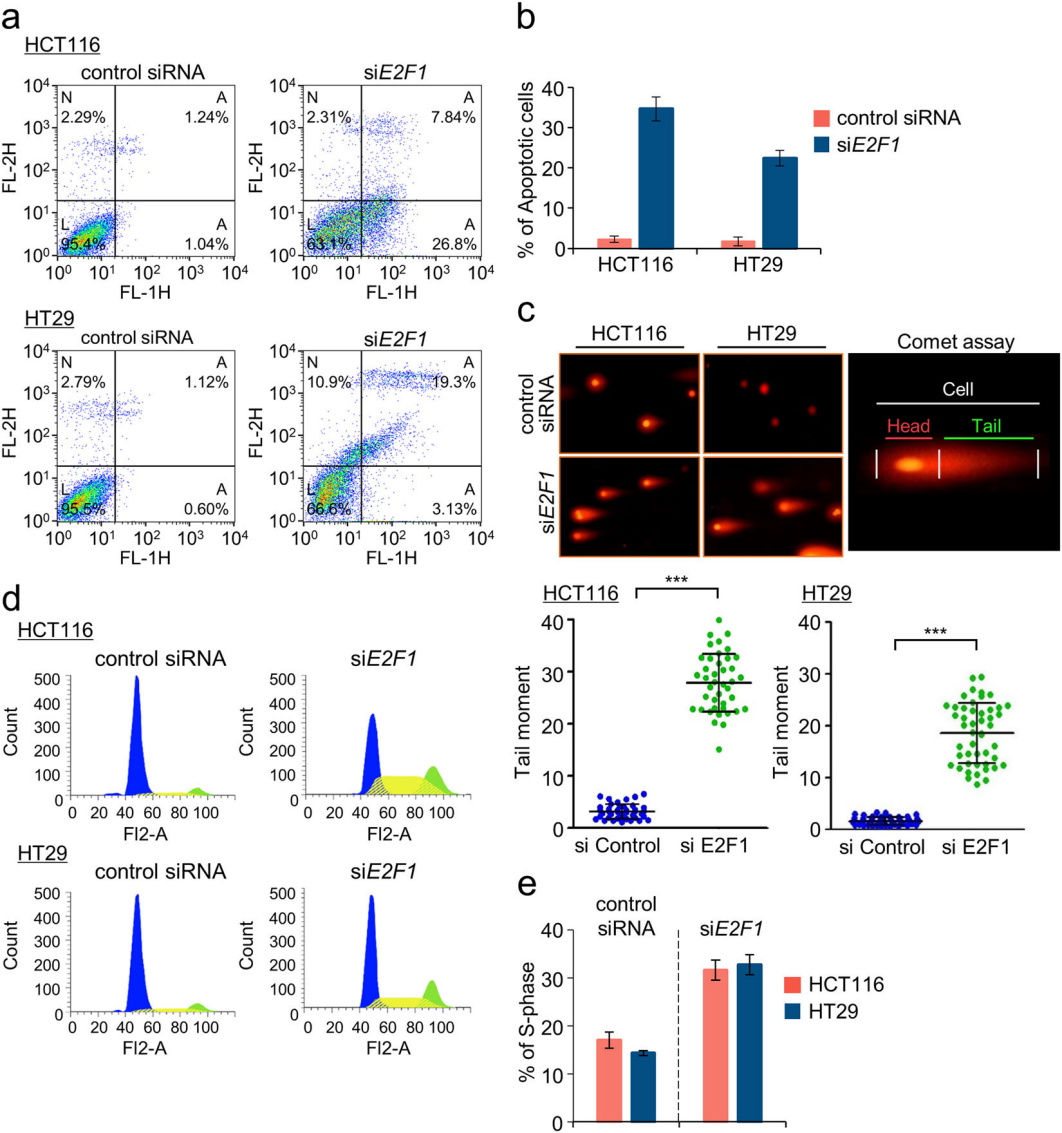
E2F1 knockdown induces cell death in human colon cancer cells. **a** Apoptosis analysis of HCT116 and HT29 cells via flow cytometry. Colon cancer cells were incubated with siRNA in serum-free medium. The proportion of apoptotic cells was quantified using FITC-conjugated annexin V (1.5 μg/ml) and PI (20 μg/ml). Scatter plots illustrate the distribution of FITC-annexin V and PI staining for siControl- and si*E2F1*-transfected cells. The cells are classified as “live-cell” (bottom left), “early apoptotic-cell” (bottom right), “late apoptotic-cell” (top right) and “necrotic-cell” (top left). **b** Quantitative analysis of apoptotic cells in response to *E2F1* knockdown. The bar graph shows the total percentages of early and late apoptotic cells determined by flow cytometry. FACS data was quantified using FlowJo software. Error bars denote the mean ± SD (*n* = 3). **c** The degree of DNA damage was measured via comet assay using Nikon Ti-E fluorescence microscopy. The lengths of fifty independent cell comet tails per sample were analyzed with Casp software (1.2.3beta2) (bottom). Three independent experiments were performed, and tail moments were measured (tail length × % of DNA in the tail). Error bars denote the mean ± SD (*n* = 3). ***P < 0.001 (Student’s *t*-test) indicates significance compared with siControl-treated cells. **d** Cell cycle profiles of human colon cancer cells treated with si*E2F1* as characterized by flow cytometry. **e** Analysis of cell cycle progression by flow cytometry after si*E2F1* transfection. The percentages of siControl-transfected and E2F1-deficient cells in S-phase were quantified with FlowJo software. Three independent experiments were performed. Error bars denote the mean ± SD (*n* = 3)

To determine whether the E2F1-dependent expression of HR factors affects cell viability and proliferation through HR progression, we knocked down the *E2F1* gene for 48 h with small interfering RNA (siRNA), and the cells were stained with PI and FITC-Annexin-V antibody. Apoptotic and necrotic cells were then identified via flow cytometric analysis. Within HCT116 and HT29 cells in the presence or absence of E2F1, we classified cell death processes observed by flow cytometry into four groups: necrotic (quadrant 1), late apoptotic (quadrant 2), early apoptotic (quadrant 3), and live (quadrant 4) processes (Fig. 2a). The proportion of apoptotic cells was increased in cells lacking E2F1 (6.60% of HCT116 cells were in the late apoptotic stage and 25.76% were in the early apoptotic stage; 18.18% of HT29 cells were in the late apoptotic stage, and 2.53% were in the early apoptotic stage) compared with that in normal control cells. *E2F1* knockdown, therefore, increases apoptosis in colon cancer cells compared with that in normal control cells (Fig. 2a, b). These results suggest that low levels of HR factors caused by the depletion of E2F1 can result in the accumulation of various types of DNA breaks and lesions in colon cancer cells resulting from an incomplete HR pathway.

To investigate whether E2F1 expression is required to enhance DNA break repair, we performed comet assays with HCT116 and HT29 cells (Fig. 2c). Notably, the knockdown of *E2F1* increased the number of cells exhibiting DNA tail moments. In addition, the DNA tails in HCT116 and HT29 cells were longer than that those in normal control cells by approximately 7.4- and 6.5-fold, respectively, indicating that the absence of E2F1 during cell progression delays DSB repair and leads to DNA fragmentation following cell death (Fig. 2c). In addition, cell growth rates after *E2F1* knockdown gradually decreased over a period of 3 days, and the percentage of surviving cells was reduced to 30.4% in HCT116 cells and 25.3% in HT29 cells compared with that of normal control cells (Supplementary Fig. S2). We have also provided evidence that downregulation of HR proteins by *E2F1* knockdown may cause serious defects in DNA repair and cell cycle progression within HCT116 and HT29 cells. Thus, our results suggest that colon cancer cells continually maintain an abundant level of HR factors and that these HR factors quickly respond to DSBs, thereby promoting cell cycle progression, cellular proliferation, and cell viability.

### E2F1 is required for cell progression and G2-to-M transition

To determine whether E2F1 is involved in DNA replication in colon cancer cells, we conducted the BrdU cell proliferation assay following the transfection of cells with si*E2F1* (Supplementary Fig. S3a, b). *E2F1* knockdown in cancer cells caused cell cycle arrest at the S/G2 phase, leading to DNA gaps and subsequently inhibiting cellular proliferation (Fig. 2d, e; Supplementary Fig. S3a, b). Furthermore, the proportion of cells in S/G2 phase increased by ~2.40-fold in si*E2F1*-transfected HCT116 cells and by 2.92-fold in siE2F1-transfected HT29 cells when compared with that in siControl-transfected cells, as determined by flow cytometry (Fig. 2d, e). Thus, aberrant replication caused by the absence of E2F1 leads to an increase in the number of DNA gaps, which results in activation of the G2/M checkpoint^35^.

### Colon cancer cells require E2F1 to promote RAD51 foci formation

**Fig. 3:**
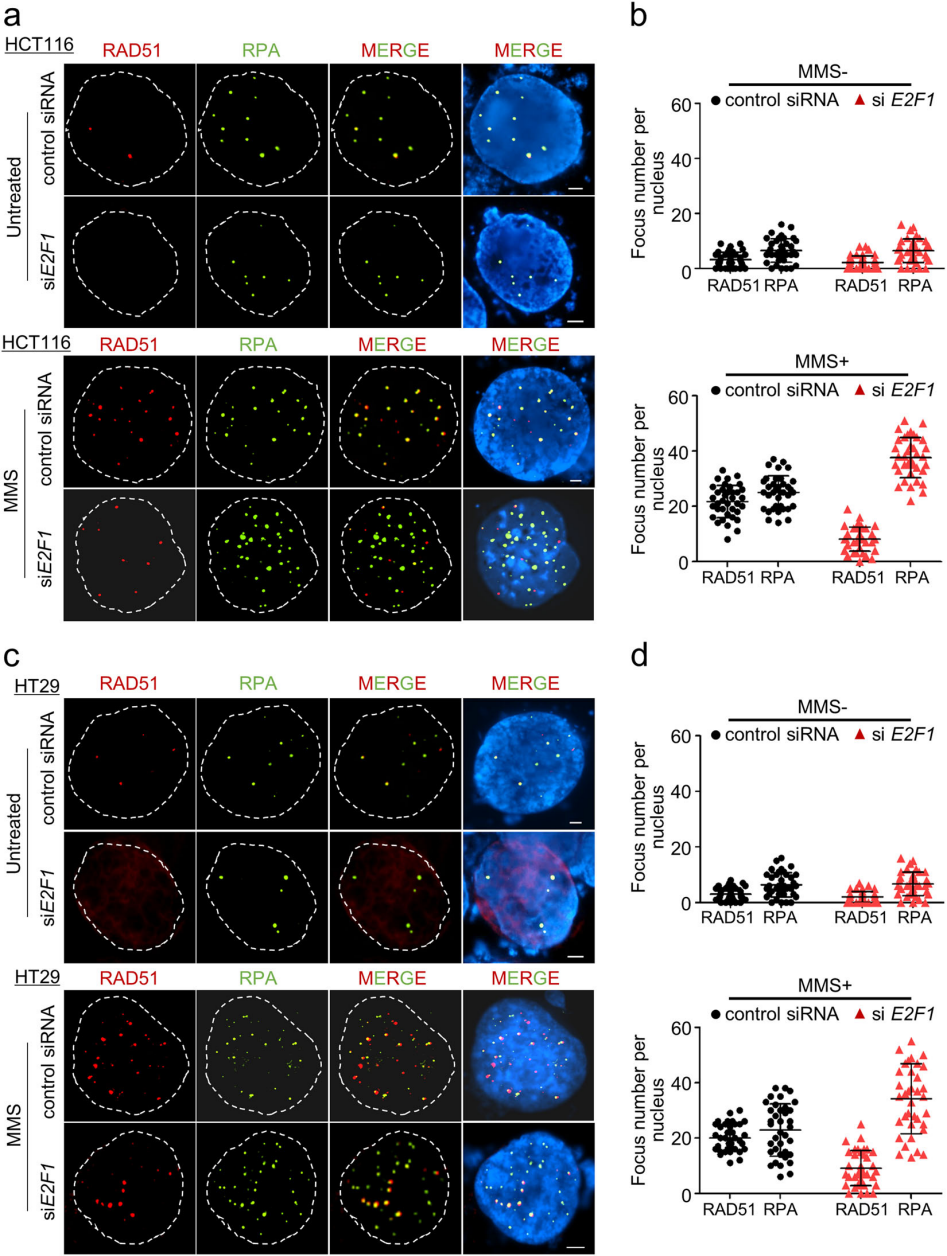
Recruitment of HR factors to DNA lesions in human colon cancer cells. **a**, **c** Representative images of RAD51 and RPA foci in HCT116 and HT29 cells under the indicated conditions. The cells were imaged after transfection with siControl or si*E2F1* for 48 h. The cells under each condition were treated with MMS (2 mM) and immunostained with anti-RPA and anti-RAD51 antibodies. Scale bars, 2.5 μm. **b**, **d** Quantitative analysis of foci formed in response to *E2F1* knockdown. The numbers of foci in each condition were quantified. The scatter plots illustrate the number of RAD51 and RPA foci per individual cell (mean ± SD), and a minimum of 30 nuclei were counted for each experiment. The number of foci per nucleus was analyzed using Prism5 software

To determine whether colon cancer cells require E2F1 to promote HR activity to induce DNA gap processing and complete DNA replication, we examined the formation of RAD51 foci and RPA foci following the treatment of colon cancer cells with si*E2F1*. RAD51 and RPA foci were observed in siControl-treated cells, with an average of 3.00 RAD51 and 6.35 RPA foci per HCT116 cell nucleus. Within HT29 cells, an average of 3.29 RAD51 and 6.54 RPA foci were observed. In addition, similar numbers of foci were observed following *E2F1* knockdown; specifically, the average numbers of RAD51 and RPA foci per HCT116 cell nucleus were 2.02 and 6.72, respectively, and the average numbers of RAD51 and RPA foci per HT29 cell nucleus were 2.2 and 6.45, respectively (Fig. 3a–d).

To further investigate the role of E2F1 in response to DNA breakage, we induced DNA damage with MMS, which causes DSBs by inducing replication blocks and base mispairing. We first optimized the concentration of MMS required to induce DSBs with minimal cytotoxicity within human cancer cell cultures. We examined the effects of MMS at the following concentrations: 1, 2, 3, and 6 mM (Supplementary Fig. S4a) and determined that 2 mM was the optimal concentration of MMS that induced DSBs without causing significant cytotoxicity (Supplementary Fig. S4). Furthermore, in response to DNA damage, the average numbers of RAD51 and RPA foci per nucleus increased to 21.72 and 25.00, respectively, in HCT116 cells and 20.08 and 22.86, respectively, in HT29 cells in the presence of E2F1. Interestingly, when E2F1 was depleted, the number of RPA foci did not change significantly compared with that observed when E2F1 was present in HCT116 and HT29 cells. However, the depletion of E2F1 caused the number of RAD51 foci to dramatically decrease by 62.6% and 54.7% in HCT116 and HT29 cells, respectively (Fig. 3a–d). These results suggest that E2F1 plays a role in the recruitment of DNA repair factors to DNA breaks. Moreover, E2F1 deficiency may impair the formation of RAD51 foci at ssDNA or sites of DNA damage by inhibiting the binding between RAD51 and ssDNA, thereby preventing the expression of RAD51 and interrupting RAD51-regulated strand exchange through inhibiting RAD51-ssDNA nucleofilament formation. For these reasons, ssDNA produced by DSBs or stalled replication forks were not repaired, and RPA foci then accumulated on the unrepaired ssDNA species. Thus, E2F1 may direct the HR-mediated DNA repair pathway, which is important for maintaining genome stability in human colon cancer cells.

### E2F1 localizes to DNA damage sites

**Fig. 4:**
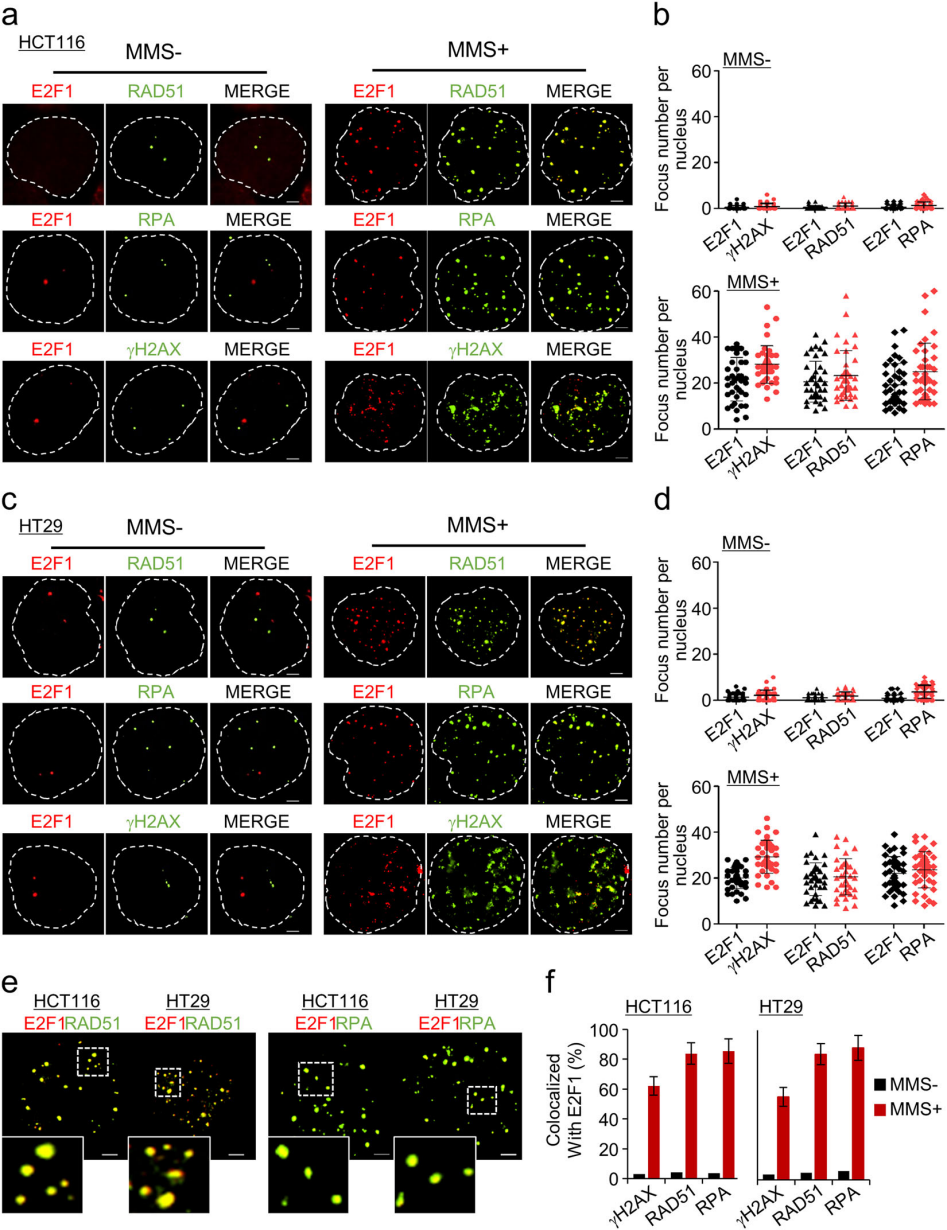
E2F1 localizes to DSB sites. **a**, **c** Representative images illustrating E2F1, RAD51, RPA, and γH2AX foci in HCT116 and HT29 cells in the presence or absence of MMS (2 mM). The cells were immunostained with anti-E2F1, anti-RAD51, anti-RPA, and anti-γH2AX antibodies. Scale bar, 2.5 μm. **b**, **d** Quantification of foci per nucleus in response to MMS. The scatter plots show the numbers of foci containing each protein per individual cell (mean ± SD). A minimum of 40 nuclei were counted for each experiment. The number of foci per nucleus was analyzed using Prism5 software. **e** Magnified image corresponding to the white dotted line of colocalized foci. **f** Quantification of the numbers of foci containing proteins colocalized with E2F1 following treatment with MMS. Error bars denote the mean ± SD

E2F1 interacts with MRE11 and localizes near DNA replication origins^36^. To determine whether E2F1 localizes to sites of DNA damage, we investigated the colocalization of endogenous E2F1 and γH2AX, which is used to detect DNA DSBs, in human colon cancer cells by immunofluorescence imaging. Following the treatment of colon cancer cells with agents that induce DNA breaks, E2F1 and γH2AX foci partially colocalized (Fig. 4a–d). In support of this finding, we observed the formation of foci containing E2F1, RAD51, and RPA bound to exposed ssDNA gaps or at DNA break sites. Interestingly, within both HCT116 and HT29 cells, 83% of the observed foci consisted of E2F1 colocalized with RAD51 and RPA (Fig. 4e, f). Furthermore, immunoprecipitation revealed that E2F1 physically interacts with RAD51 and RPA (Supplementary Fig. S5). Taken together, these findings imply that E2F1 forms foci at DNA break sites following DNA damage and is thus associated with DNA repair. Moreover, E2F1 appears to collaborate with DNA repair proteins at DNA break sites to mediate DNA repair and complete DNA replication.

### The depletion of E2F1 promotes the accumulation of DNA breaks

**Fig. 5:**
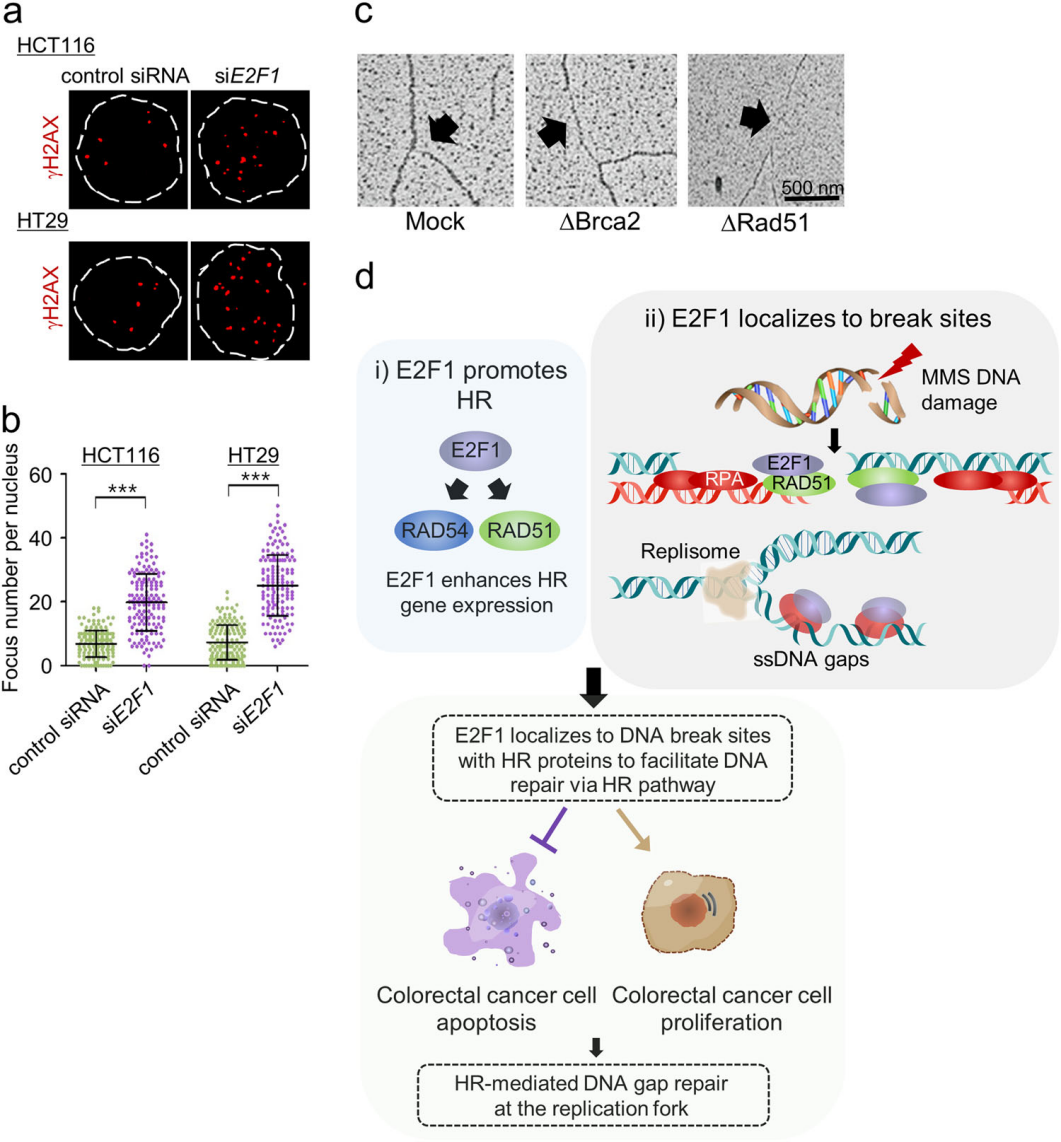
Depletion of E2F1 induces DSBs and ssDNA gaps. **a** Representative microscopic images of cells showing γH2AX foci in response to *E2F1* knockdown. The nuclear perimeter was stained with DAPI (4′,6-diamidino-2-phenylindole). **b** Quantitative analysis of foci formed in response to *E2F1* knockdown. The scatter plots illustrate the number of γH2AX foci per individual cell (mean ± SD), and a minimum of 150 nuclei were counted for each experiment. The number of foci per nucleus was analyzed using Prism5 software. ***P < 0.001 (Student’s *t*-test) indicates significance compared with siControl-treated cells. **c** Electron microscopic images showing replication forks isolated from *Xenopus laevis* (modified from Kolinjivadi et al. (2017)^51^). Following BRCA2 or RAD51 depletion, ~500 nucleotide-long ssDNA gaps are observed, but these gaps are not observed in control cells. **d** The proposed model by which E2F1-mediated HR factors maintain genomic integrity. When E2F1 activates HR factors by binding at their promoters, HR responds efficiently to induce DNA replication and DNA repair. E2F1 effectively localizes to DNA break sites, where it interacts with regulatory proteins involved in the HR pathway. However, E2F1 deficiency can cause replication fork collapse or the accumulation of DSBs or ssDNA gaps at S/G2 transition, inducing cell cycle arrest in G2/M phase or cell death

Immunofluorescence staining of γH2AX was employed to estimate the number of DSBs in cells treated with siControl and si*E2F1*. When the H2AX histone is phosphorylated at its serine 139 residue, it is designated γH2AX and used to detect DSBs^37^. Among HCT116 cells, the number of γH2AX foci per cell was 6.82, while among *E2F1* knockdown cells, the number of γH2AX foci per cell increased by 2.9-fold when compared with that for siControl-treated cells (Fig. 5a, b). In addition, control HT29 cells contained ~7.25 γH2AX foci per cell, whereas the γH2AX foci per *E2F1* knockdown cell was increased by 3.45-fold compared with that per siControl-treated cell (Fig. 5a, b). Western blot analysis revealed that HCT116 *E2F1* knockdown cells were unable to efficiently repair DNA and that γH2AX accumulated at higher levels than siControl-treated HCT116 cells following DNA damage (Supplementary Fig. S6). Furthermore, following MMS treatment, γH2AX was induced in both siControl-treated and *E2F1* knockdown cells, although the expression levels of γH2AX in *E2F1* knockdown cells were dramatically increased (Supplementary Fig. S6). Moreover, while *E2F1* knockdown following 1 h of si*E2F1* treatment reduced RAD51 levels, RAD51 expression persisted for up to 6 h within siControl-treated cells. Consistent with the results above, *E2F1* knockdown cells accumulated remarkably more DSBs prior to treatment with MMS than normal cells (Supplementary Fig. S6). These results thus reveal that the absence of E2F1 leads to the accumulation of DSBs and ssDNA gaps by impairing repair systems.

## Discussion

The transcription factor E2F1 is involved in DNA replication and repair in many types of human cancers^38^. Previous studies have reported that E2F1 is expressed at high levels in most human cancers, including colon cancer and breast cancer and that E2F1 expression in an individual cell facilitates the efficient repair of DSBs and stalled replication forks^35,39^. However, the reason for high-level E2F1 expression in human cancer cells and the specific role of E2F1 in maintaining genomic integrity in cancerous cells are not clearly understood. Here, we reveal a mechanism for the regulatory function of E2F1 in the DNA repair system that involves genomic instability and programmed cell death. Dynlacht and colleagues suggested the possible functions of E2F1 in gene regulation through ChIP experiments^40^. They also suggested that E2F1 targets diverse groups of genes related to DNA repair, replication, cell cycle regulation, and G2/M transition and physically interacts with promoters associated with HR and replication-related genes^37^. Furthermore, it has been reported that E2F1 localizes to DSB sites, where it physically interacts with NBS1 under conditions of DNA damage-induced stress^41^, implying that E2F1 is involved in DSB repair via the formation of a DNA repair complex with HR-related proteins.

HR is a conserved process that takes place in many different species, including mammals, plants, and yeast. This process plays an essential role in genome stability^42,43^. Specifically, HR promotes the progression of replication forks and the repair of DSBs and ssDNA gaps. Replication fork progression encounters interference from various endogenous and exogenous stresses, ultimately causing replication forks to stall, break, or collapse, thereby inducing a response to DNA damage^44,45^. DNA replication failure is thus responsible for the generation of genomic instability and the accumulation of mutations. Furthermore, in many organisms, delayed DNA replication is related to chromosome fragmentation, which is induced by DSBs or ssDNA gaps^46,47^. Moreover, impaired fork progression may interrupt the completion of DNA replication, which then leads to irregular chromosome segregation and the accumulation of mutations within the genome that can induce tumorigenesis^48^. Thus, genomic instability is a hallmark of tumor cells. The progression of abnormal HR pathways has been reported in cancer-causing conditions or during tumor development, which leads to an increased risk of genetic instability. More precisely, regulation of the recombinases RAD51, RAD52, and RAD54 has been shown to affect genomic integrity and regulation of the cell cycle. Our results demonstrated that cells lacking RAD51 experienced cell cycle arrest in the G2/M phase and the accumulation of DNA breaks, apoptosis, and unrepaired ssDNA, implying that the HR factor-dependent pathway is likely essential for the maintenance of genomic integrity^35^.

Recent studies have suggested the function of E2F1 at DNA damage sites and asserted that E2F1 promotes the recruitment of DNA repair proteins to DSB sites^34,41^. However, the mechanism by which E2F1 recruits these proteins and promotes DNA repair is not clearly understood. Therefore, we hypothesized that E2F1 forms foci with DNA repair factors at break sites. Interestingly, we found that E2F1 rapidly accumulates at break sites following the induction of DNA damage with MMS and that a large portion of these E2F1 foci contain DNA repair proteins, specifically RAD51 and RPA. These findings suggest that E2F1 regulates the localization of DNA repair proteins to damaged sites. Alternatively, E2F1 may direct DNA repair signaling via ATM/ATR at break sites^49^. Furthermore, our results show that E2F1 depletion affects the expression of RAD51, a core factor in the HR pathway, at the transcriptional level. RAD51 plays a crucial role in DNA break repair, and RAD54 stabilizes RAD51-ssDNA filaments in the synapsis phase and stimulates DNA strand exchange^50^. Moreover, cells that contain inactive HR proteins attempt to mediate a general homology search of homologous chromatids, leading to serious defects in DNA repair and the cell cycle. In addition to of its role in regulating the expression of genes associated with DNA repair, E2F1 may also play a central role in DSB repair by promoting the direct recruitment of HR proteins or activating HR proteins at sites of DNA breaks^41^.

To determine which of these mechanisms is employed by E2F1, we investigated RAD51 and RPA foci formation following treatment of the cells with MMS. In response to DNA damage, the proportion of foci containing RPA was dramatically increased, indicating that the presence of numerous ssDNAs generated through DNA end processing is required for both microhomology-mediated end joining and HR and that these ssDNAs are stabilized by the binding of RPA at DSB sites. In contrast, E2F1 depletion reduced RAD51 focus formation as well as the proportion of foci in which RAD51 and RPA were colocalized (Fig. 3; Supplementary Fig. S7). These results suggest that a reduction in the number of foci containing RAD51 bound to ssDNA during cell progression may cause irregular strand pairing and sequence homology searching, which regulates the invasion of DNA strands by RAD51-ssDNA nucleofilaments that repair DSBs and results in the accumulation of ssDNA gaps (Fig. 5c). Furthermore, E2F1 depletion may have suppressed the HR pathway by interfering with RAD51-mediated DNA recombination and the accumulation of ssDNA gaps, impairing DNA damage repair mechanisms in colon cancer cells (Fig. 5d). Further experimental studies will examine the functional role of E2F1 in RAD51-dependent DSB repair pathways at break sites and determine the roles of E2F1 in DNA damage independent of gene regulation, including its roles in cell cycle arrest, apoptosis, and cellular senescence.

In addition to E2F1, elevated levels of HR proteins in colon cancer cells may facilitate HR, fork reversal, and PRR, which is the most efficient pathway for ssDNA gap repair during DNA replication and simultaneously promotes the repair of damaged DNA via a sister template after DNA replication. Alternatively, HR proteins may cooperative to prevent additional resection of DNA breaks that would yield ssDNA gaps in colon cancer cells. These mechanisms preserve efficient cell cycle progression and genomic integrity in colon cancer cells by using high-fidelity HR pathways to repair DSBs and ssDNA gaps to overcome the replication stress-induced delay of cell proliferation. In human colorectal cancer cells, defective HR factors seriously impair cellular progression and cell death (Fig. 5d). Therefore, our data suggest a regulatory system for HR mediated by E2F1. In addition, E2F1 may independently serve as a biomarker or novel gene target for cancer therapy.

## Supplementary information

Supplemental Information (supple figures and table)

## References

[1] Hélène G, Tatiana GM, Andrés A (2015). Replication stress and cancer. Nat. Rev. Cancer.

[2] Zhang J, Dai Q, Park DK, Deng X (2016). Targeting DNA replication stress for cancer therapy. Genes.

[3] Macheret M, Halazonetis TD (2015). DNA replication stress as a hallmark of cancer. Ann. Rev. Pathol..

[4] Iwamoto M (2004). Overexpression of E2F-1 in lung and liver metastases of human colon cancer is associated with gene amplification. Cancer Biol. Ther..

[5] Puigvert JC, Sanjiv K, Helleday T (2016). Targeting DNA repair, DNA metabolism and replication stress as anti-cancer strategies. Febs. J..

[6] Tenca P (2007). Cdc7 is an active kinase in human cancer cells undergoing replication stress. J. Biol. Chem..

[7] Marchetti MA (2002). A single unbranched S-phase DNA damage and replication fork blockage checkpoint pathway. Proc. Natl Acad. Sci. USA.

[8] Rothstein R, Michel B, Gangloff S (2000). Replication fork pausing and recombination or “gimme a break”. Genes Dev..

[9] Kim KP (2010). Sister cohesion and structural axis components mediate homolog bias of meiotic recombination. Cell.

[10] Yoon SW (2016). Meiotic prophase roles of Rec8 in crossover recombination and chromosome structure. Nucleic Acids Res..

[11] Hong SG (2013). The logic and mechanism of homologous recombination partner choice. Moll. Cell.

[12] Kotsantis P, Petermann E, Boulton SJ (2018). Mechanisms of oncogene-induced replication stress: jigsaw falling into place. Cancer Disco..

[13] Champeris TS (2014). Licensing of DNA replication, cancer, pluripotency and differentiation: An interlinked world?. Semin. Cell Dev. Biol..

[14] Chong JL (2009). E2f1-3 switch from activators in progenitor cells to repressors in differentiating cells. Nature.

[15] Rayman JB (2002). E2F mediates cell cycle-dependent transcriptional repression in vivo by recruitment of an HDAC1/mSin3B corepressor complex. Genes Dev..

[16] Wu X, Levine AJ (1994). p53 and E2F-1 cooperate to mediate apoptosis. Proc. NatI Acard. Sci. USA.

[17] Stanelle J, Stiewe T, Theseling CC, Peter M, Pützer BM (2002). Gene expression changes in response to E2F1 activation. Nucleic Acids Res..

[18] Humbert PO (2000). E2f3 is critical for normal cellular proliferation. Genes Dev..

[19] Wu L (2001). The E2F1-3 transcription factors are essential for cellular proliferation. Nature.

[20] DeGregori J, Kowalik T, Nevins JR (1995). Cellular targets for activation by the E2F1 transcription factor include DNA synthesis- and G1/S-regulatory genes. Mol. Cell Biol..

[21] Bing R (2002). E2F integrates cell cycle progression with DNA repair, replication, and G2/M checkpoints. Genes Dev..

[22] Gorgoulis VG (2002). Transcription factor E2F-1 acts as a growth-promoting factor and is associated with adverse prognosis in non-small cell lung carcinomas. J. Pathol..

[23] Huang CL (2007). E2F1 overexpression correlates with thymidylate synthase and survivin gene expressions and tumor proliferation in non small-cell lung cancer. Clin. Cancer Res..

[24] Suzuki T (1999). Expression of the E2F family in human gastrointestinal carcinomas. Int. J. Cancer.

[25] Nevins JR (2001). The Rb/E2F pathway and cancer. Hum. Mol. Genet..

[26] Han S (2003). E2F1 expression is related with the poor survival of lymph node-positive breast cancer patients treated with fluorouracil, doxorubicin and cyclophosphamide. Breast Cancer Res. Treat..

[27] Richardson C, Stark JM, Ommundsen M, Jasin M (2004). Rad51 overexpression promotes alternative double-strand break repair pathways and genome instability. Oncogene.

[28] Raderschall E (2002). Elevated levels of Rad51 recombination protein in tumor cells. Cancer Res..

[29] Kato M (2000). Identification of Rad51 alteration in patients with bilateral breast cancer. J. Hum. Genet..

[30] Schild D, Wiese C (2010). Overexpression of RAD51 suppresses recombination defects: a possible mechanism to reverse genomic instability. Nucleic Acid Res..

[31] Tennstedt P (2013). RAD51 overexpression is a negative prognostic marker for colorectal adenocarcinoma. Int. J. Cancer.

[32] Choi EH, Yoon S, Hahn Y, Kim KP (2017). Cellular dynamics of Rad51 and Rad54 in response to postreplicative stress and DNA damage in HeLa cells. Mol. Cells.

[33] Rubin SM (2005). Structure of the Rb C-terminal domain bound to E2F1-DP1: a mechanism for phosphorylation-induced E2F release. Cell.

[34] Biswas AK, Johnson DG (2012). Transcriptional and nontranscriptional functions of E2F1 in response to DNA damage. Cancer Res..

[35] Choi, E. H., Yoon, S., Park, K. S. & Kim, K. P. The homologous recombination machinery orchestrates post-replication DNA repair during self-renewal of mouse embryonic stem cells. *Sci. Rep*. **7**, 10.1038/s41598-017-11951-1 (2017). 10.1038/s41598-017-11951-1 PMC5599617 28912486

[36] Richard. SM (2001). Mre11 complex and DNA replication: linkage to E2F and sites of DNA synthesis. Mol. Cell Biol..

[37] Kuo. LJ, Yang. LX (2008). Gamma-H2AX—a novel biomarker for DNA double-strand breaks. Vivo.

[38] David E, Brigitte MP (2012). The dark side of E2F1: in transit beyond apoptosis. Cancer Res..

[39] Hine CM, Seluanov A, Gorbunova V (2008). Use of the Rad51 promoter for targeted anti-cancer therapy. Proc. NatI Acad. Sci. USA.

[40] Takahashi Y, Rayman JB, Dynlacht BD (2000). Analysis of promoter binding by the E2F and pRB families in vivo: distinct E2F proteins mediate activation and repression. Genes Dev..

[41] Chen J (2011). E2F1 promotes the recruitment of DNA repair factors to sites of DNA double-strand breaks. Cell Cycle.

[42] Choi. EH, Yoon S, Kim KP (2018). Combined ectopic expression of homologous recombination factors promotes embryonic stem cell differentiation. Mol. Ther..

[43] Hong. S, Joo JH, Yun H, Kim K (2019). The nature of meiotic chromosome dynamics and recombination in budding yeast. J. Microbiol..

[44] Lambert S, Carr AM (2005). Checkpoint responses to replication fork barriers. Biochimie.

[45] Jasin M., Rothstein R. (2013). Repair of Strand Breaks by Homologous Recombination. Cold Spring Harbor Perspectives in Biology.

[46] Branzei D, Foiani M (2010). Maintaining genome stability at the replication fork. Nat. Rev. Mol. Cell Biol..

[47] Yoon S, Choi EH, Kim JW, Kim KP (2018). Structured illumination microscopy imaging reveals synaptonemal complex and localization of replication protein A during mammalian meiosis. Exp. Mol. Med..

[48] Aguilera A, Garcia MT (2013). Causes of genome instability. Annu. Rev. Genet..

[49] Lin WC, Lin FT, Nevins JR (2001). Selective induction of E2F1 in response to DNA damage, mediated by ATM-dependent phosphorylation. Genes Dev..

[50] Yoon SW, Kim DK, Kim KP, Park KS (2014). Rad51 regulates cell cycle progression by preserving G2/M transition in mouse embryonic stem cells. Stem Cells Dev..

[51] Kolinjivadi AM (2017). Smarcal1-mediated fork reversal triggers Mre11-dependent degradation of nascent DNA in the absence of Brca2 and stable Rad51 nucleofilaments. Mol. Cell.

