# Supplementary material for: E2F1 facilitates DNA break repair by localizing to break sites and enhancing the expression of homologous recombination factors
Source: Exp Mol Med. 2019 Sep 18;51(9):106. doi: 10.1038/s12276-019-0307-2 (PMC6802646; doi:10.1038/s12276-019-0307-2)
Supplement: Supplementary file 1 — Supplemental Information (supple figures and table) [file 12276_2019_307_MOESM1_ESM.docx]

**E2F1 facilitates DNA break repairs by localizing to break sites and enhancing expression of homologous recombination factors**

Eui-Hwan Choi^1^ and Keun Pil Kim^1,^*

^1^ Department of Life Sciences, Chung-Ang University, Seoul 06974, South Korea

* To whom correspondence should be addressed.

Keun P. Kim

Department of Life Sciences
Chung-Ang University

Seoul 156-756

Korea

Email: [kpkim@cau.ac.kr](mailto:kpkim@cau.ac.kr)

Phone: 82-2-820-5792

Fax: 82-2-820-5206

**
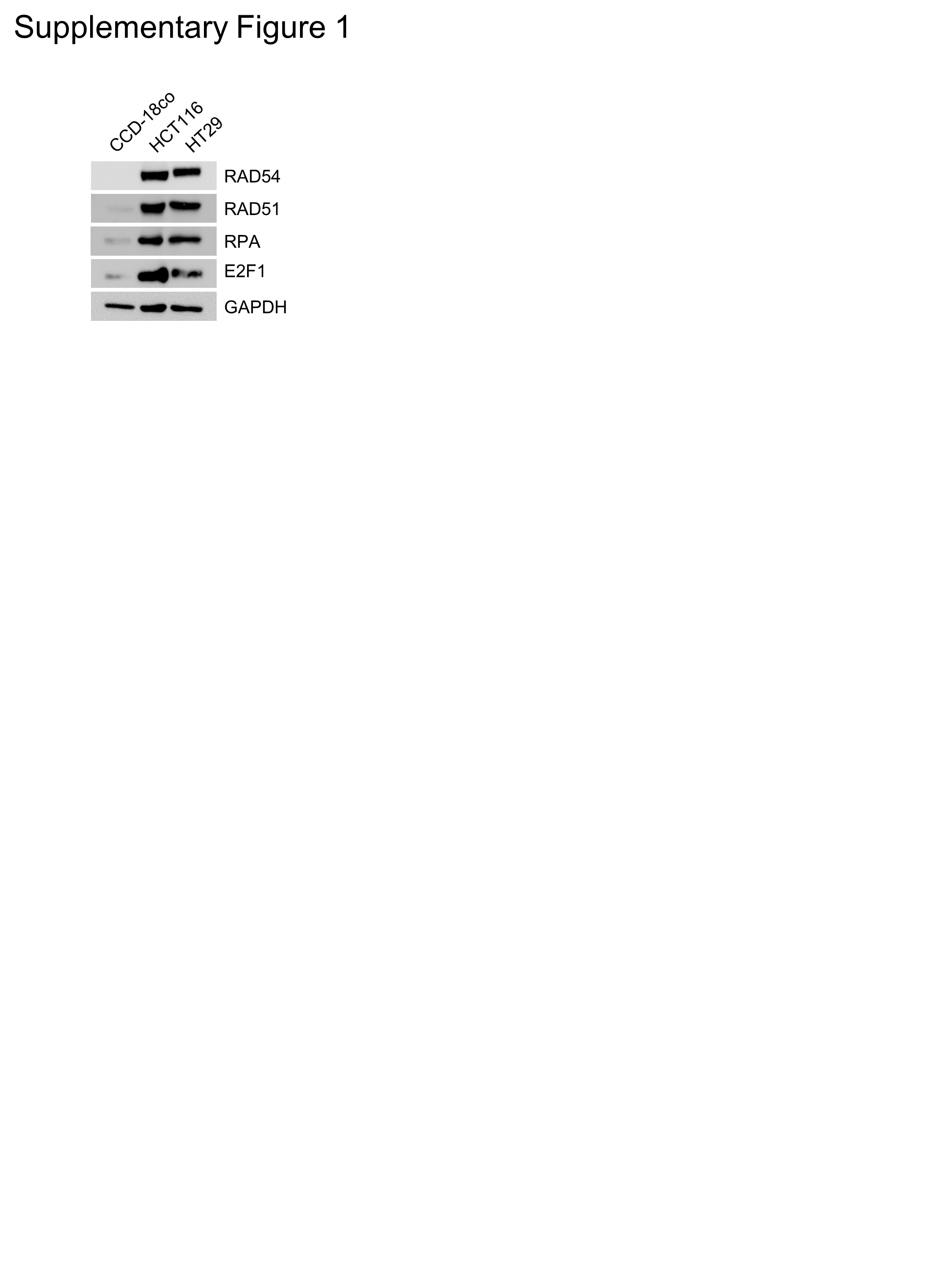
**

**Supplementary Figure 1.** **Analysis of gene expression in human colon cancer and human colon normal cells.**

Comparison of RAD54, RAD51, RPA, E2F1, and GAPDH protein expression levels by immunoblot analysis from asynchronous cells

**
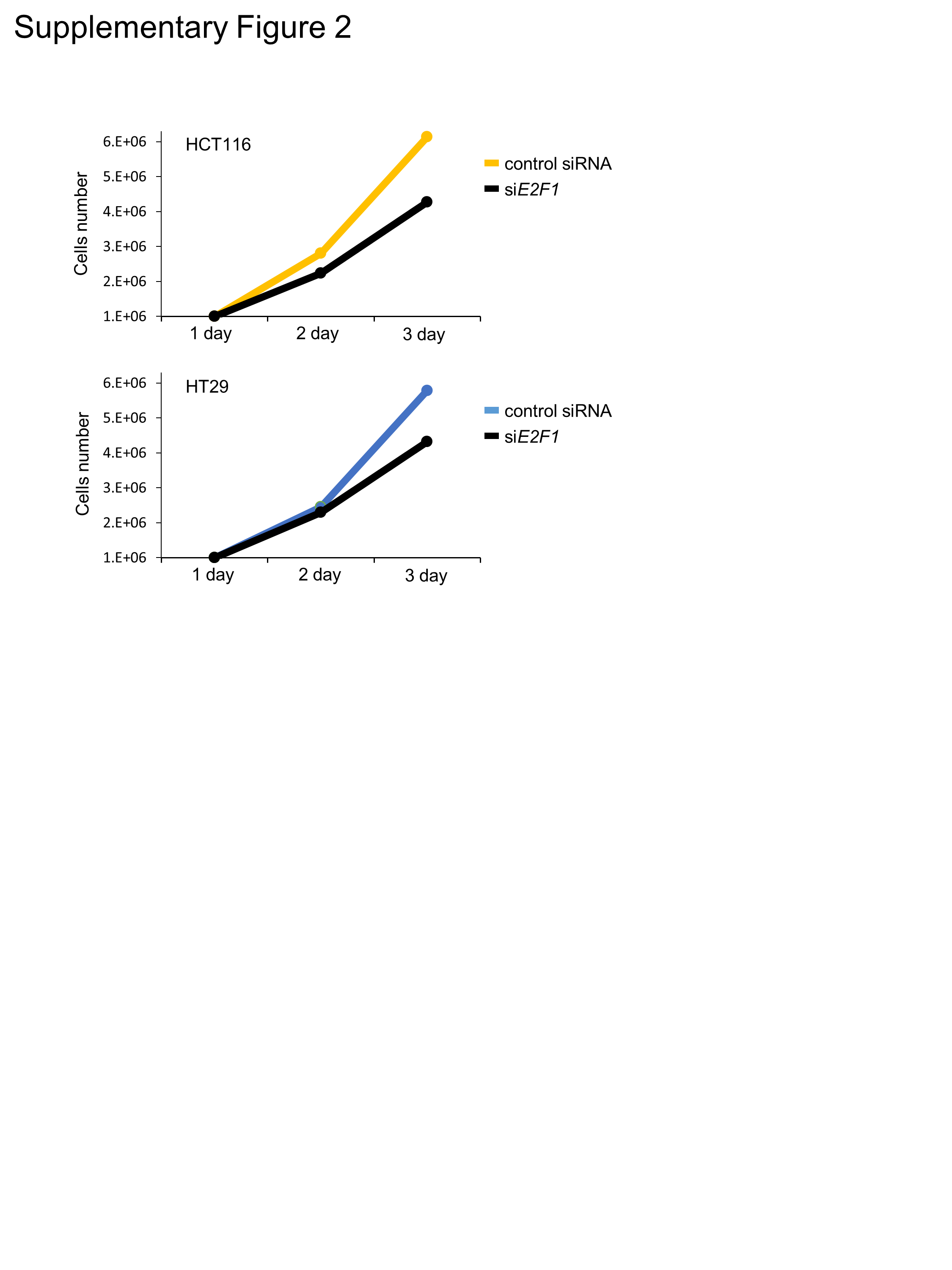
**

**Supplementary Figure 2.** **Analysis of cell growth rates after *E2F1* knockdown**

Quantification of cell numbers in human colon cancer cells. Three independent experiments were performed and analyzed cell numbers using a hemocytometer. Error bars indicate the mean ± SD (n = 3)


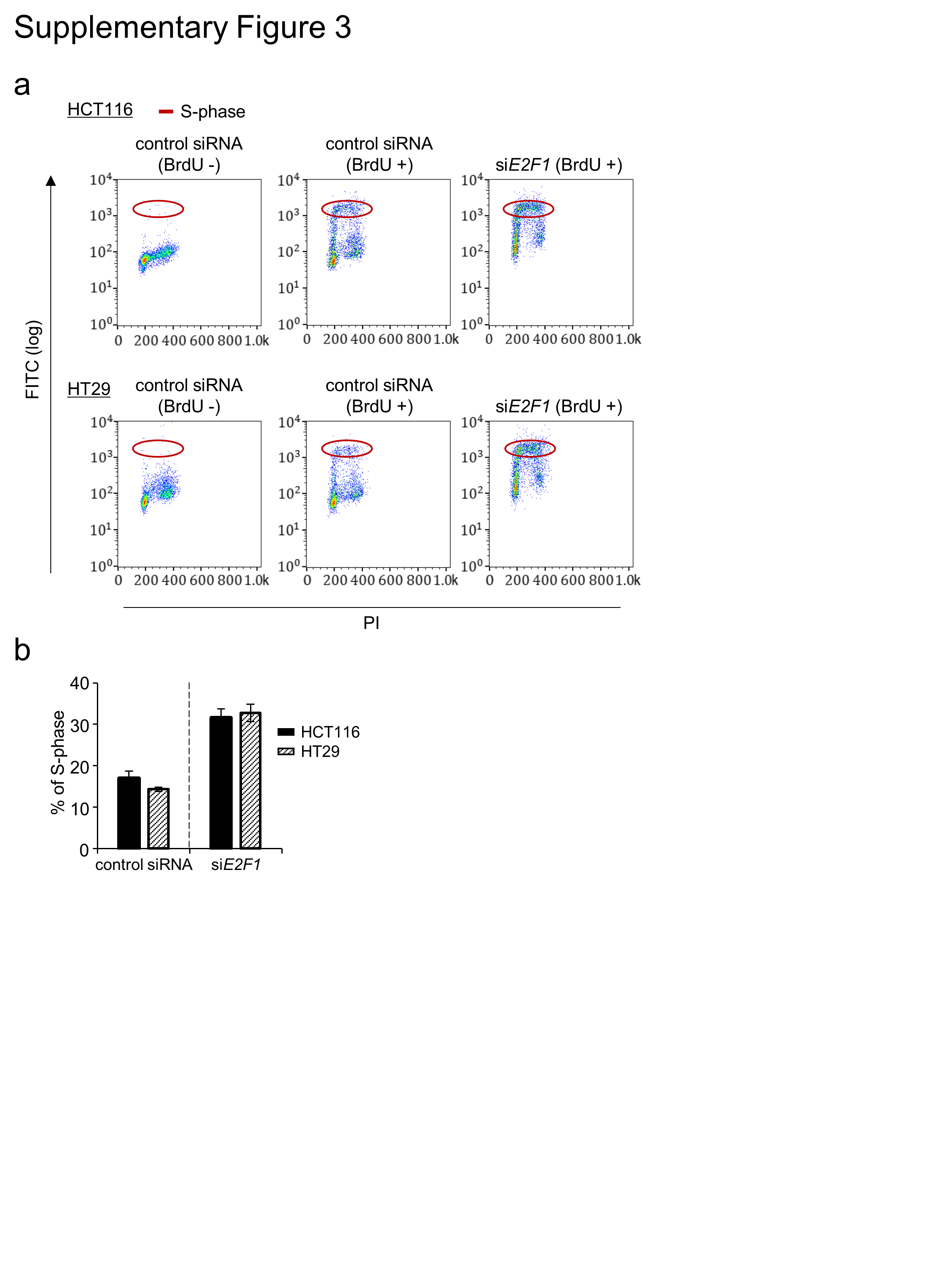


**Supplementary Figure 3.** **FACS analysis of S-phase progression after *E2F1* knockdown. a** Analysis of the cell cycle profiles after *E2F1* knockdown DNA synthesis measured by BrdU incorporation. The mean BrdU fluorescence intensity and the percentage of cells in S phase are indicated for two type of human colon cancer cell. **b** Quantitative analysis of S-phase cells in response to *E2F1* knockdown. The bar graph means total percentages of S-phase cells by flow cytometry analysis. The relative population of *E2F1* knockdown cells was quantified in the presence of BrdU using Flow Jo software. Error bars denote the mean ± SD (n = 3)


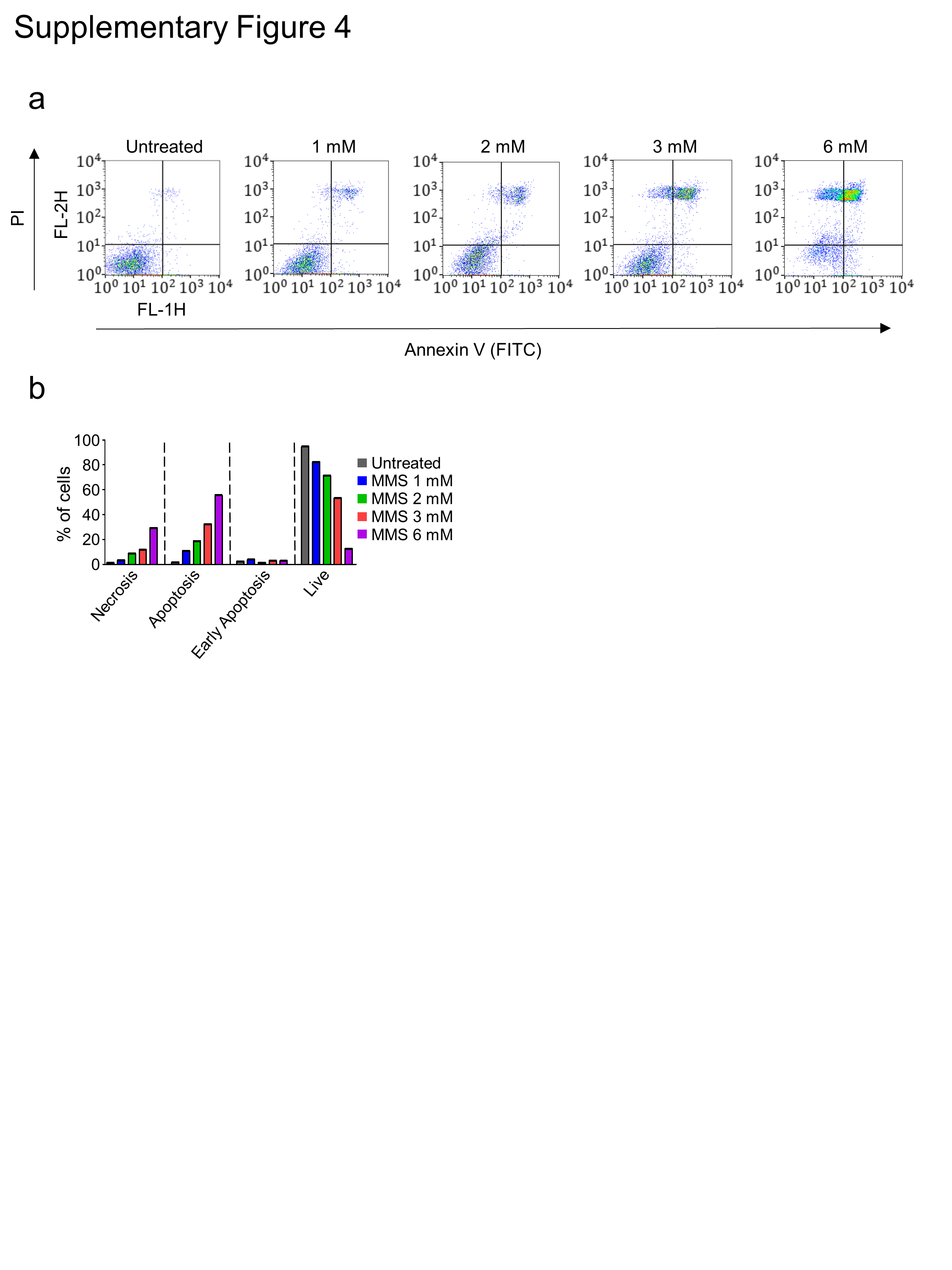


**Supplementary Figure 4.** **Analysis of apoptosis and cell viability in response to MMS-reagent. a** FACS analysis of population of apoptotic and necrotic cells on diverse concentration of HU reagent. Human colon cancer cells were treated with MMS (untreated, 1 mM, 2 mM, 3 mM, and 6 mM) as described in Materials and Methods and stained with PI. **b** The cell numbers on various MMS concentration. Human colon cancer cells were treated with MMS and the viable cells were profiled using Flow jo software.


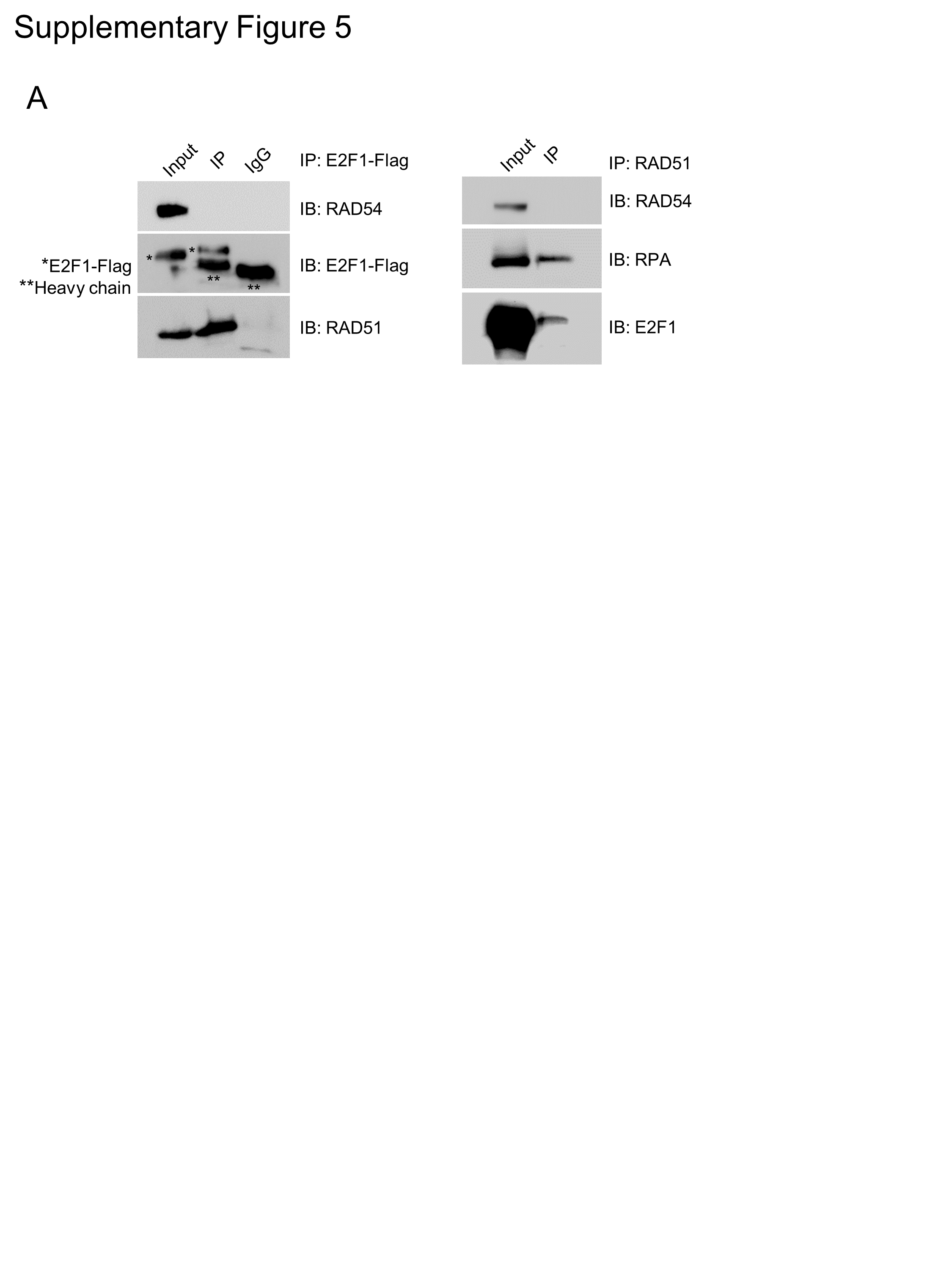


**Supplementary Figure 5. Identification of E2F1 with interacting partner.**

HCT116 cells transfected with pCMV-Flag-E2F1 were lysed and immunoprecipitated with anti-Flag antibody. The bound proteins were analyzed by immunoblot.


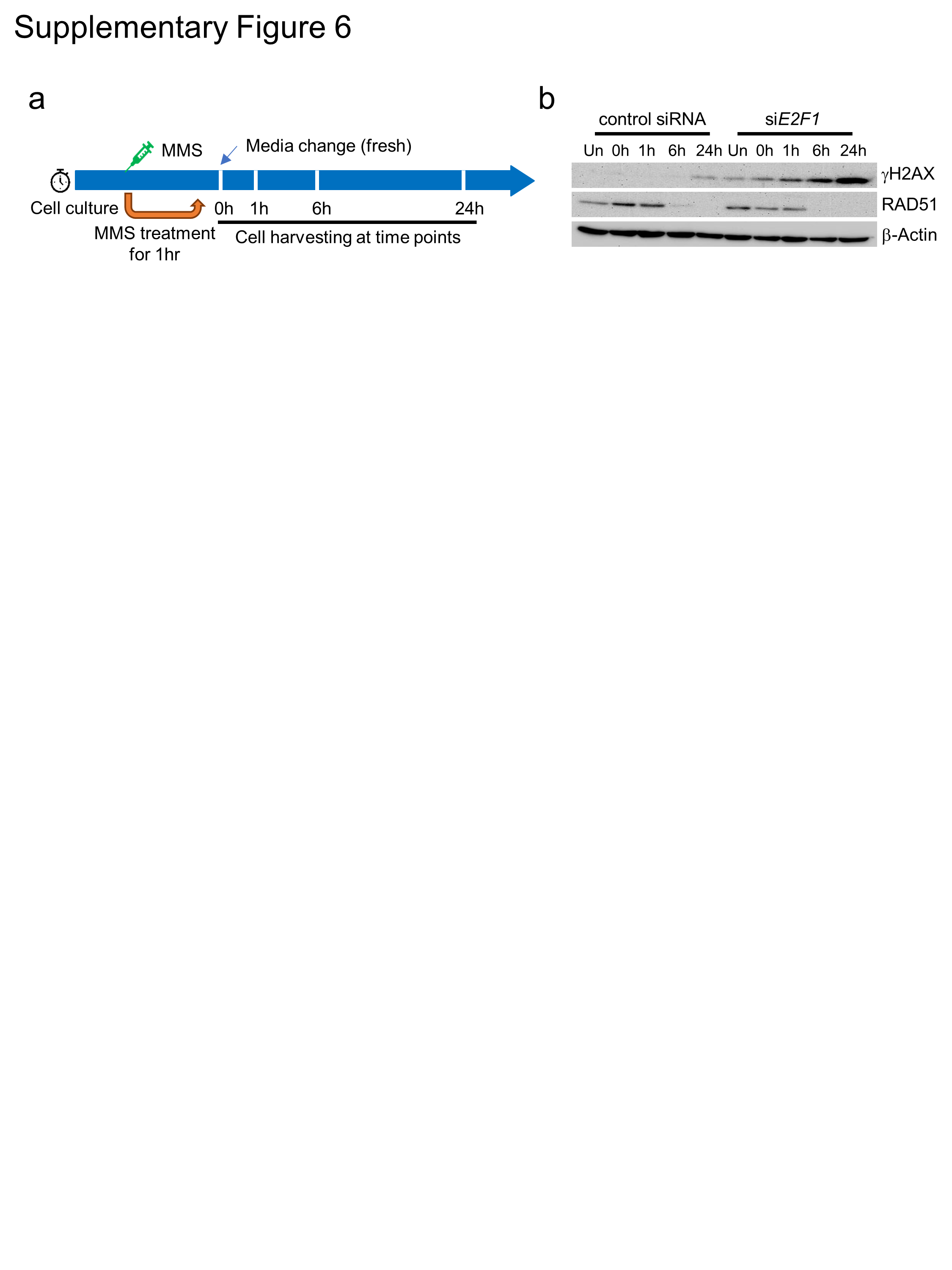


**Supplementary Figure 6. Analysis of accumulation of DNA damage response to MMS-reagent. a** Schematic of the cell recovery experiment after MMS treatment (see Materials and Methods). **b** analysis of RAD51 and γH2AX expression levels.


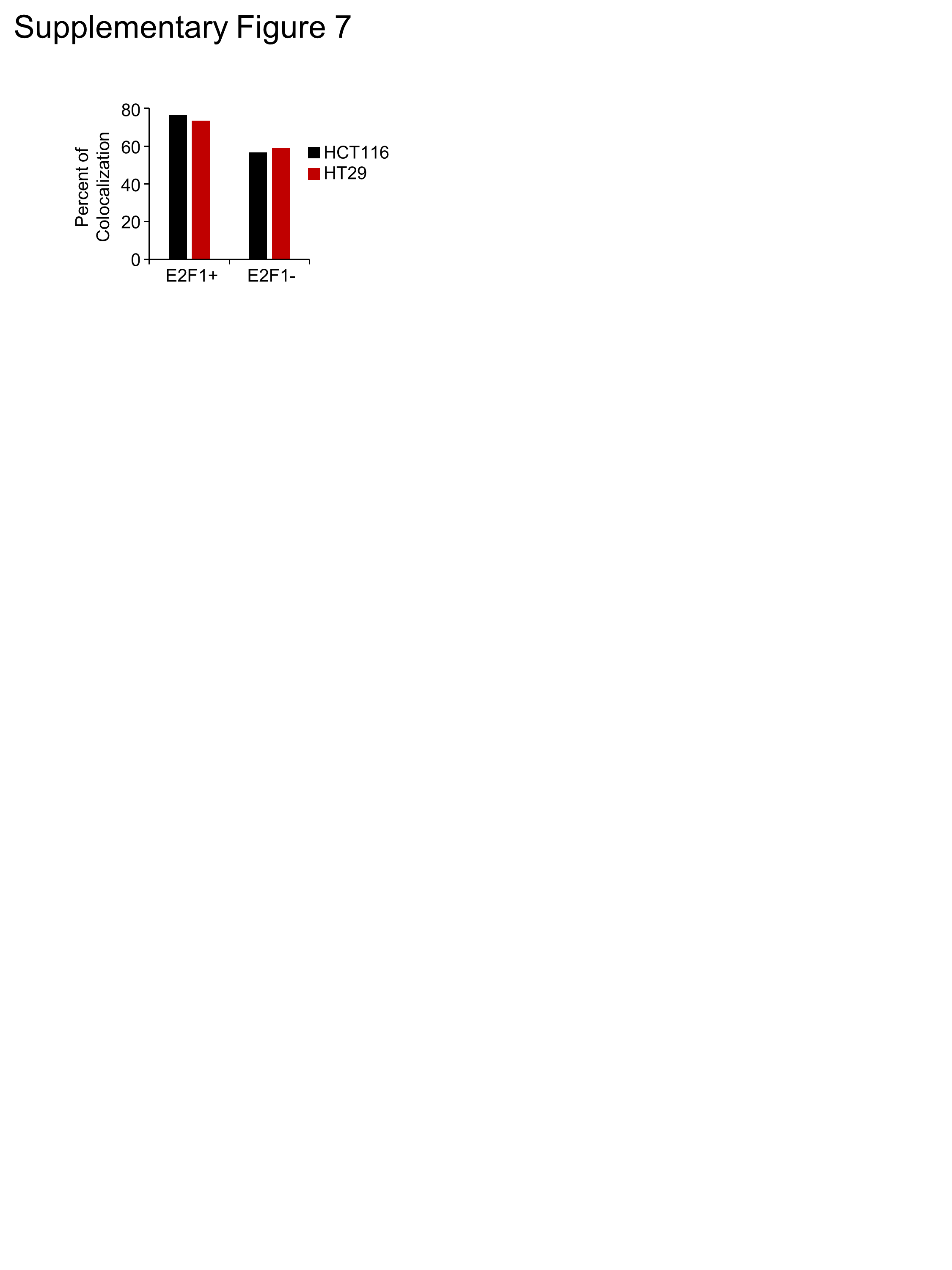


**Supplementary Figure 7.** The proportion of RAD51-RPA co-localization under the presence or absence of E2F1. Quantification of the number of RAD51 and RPA co-localization under the presence or absence of E2F1.

**Supplementary Table S1. Primer sequences used in quantitative polymerase chain reaction**

| Gene | Sequence |
| --- | --- |
| *E2F1*  *RPA2*  *RPA3*  *RAD54*  *RAD52*  *RAD51*  *PCNA*  *MRE11*  *18s rRNA* | Forward: ACT CCT CGC AGA TCG TCA TCA  Reverse: CGG GGA TTT CAC ACC TTT TCC  Forward: GGA GGC TTT TGG AGC CAA CT  Reverse: TCG AAT CCA CGG ATG CTA CG  Forward: GAT GGA GCC ACT TGA CGA GG  Reverse: CTT CAG TGT AGC TTC CTG GCA  Forward: GGT CCT ACA CTC TTA GCC GC  Reverse: TAG GAG TCA CTA GGC CAG GTT  Forward: GAG GCG CGT GTG CTA CAT TG  Reverse: TCC ACA TTC TGC TGC GTG AT  Forward: GGA GAA GGA AAG GCC ATG TA  Reverse: GGG TCT GGT GGT CTG TGT T  Forward: GCC CTC AAA GAC CTC ATC AA  Reverse: TCT GGG ATT CCA AGT TGC TC  Forward: GCG GAA TTC AGG TTT ACG GC  Reverse: TGT GTT TTC ATC ATC AAG TGC AT  Forward: GTA ACC CGT TGA ACC CCA TT  Reverse: CCA TCC AAT CGG TAG TAG CG |
